# Clinical peculiarities of tuberculosis

**DOI:** 10.1186/1471-2334-14-S1-S4

**Published:** 2014-01-08

**Authors:** Paola Piccini, Elena Chiappini, Enrico Tortoli, Maurizio de Martino, Luisa Galli

**Affiliations:** 1 Emerging Bacterial Pathogens Unit; San Raffaele Scientific Institute, Milan, Italy; 2 Department of Health Sciences, Meyer Children University Hospital, University of Florence, Florence, Italy

## Abstract

The ongoing spread of tuberculosis (TB) in poor resource countries and the recently increasing incidence in high resource countries lead to the need of updated knowledge for clinicians, particularly for pediatricians. The purpose of this article is to provide an overview on the most important peculiarities of TB in children. Children are less contagious than adults, but the risk of progression to active disease is higher in infants and children as compared to the subsequent ages. Diagnosis of TB in children is more difficult than in adults, because few signs are associated with primary infection, interferon-gamma release assays and tuberculin skin test are less reliable in younger children, *M. tuberculosis* is more rarely detected in gastric aspirates than in smears in adults and radiological findings are often not specific. Treatment of latent TB is always necessary in young children, whereas it is recommended in older children, as well as in adults, only in particular conditions. Antimycobacterial drugs are generally better tolerated in children as compared to adults, but off-label use of second-line antimycobacterial drugs is increasing, because of spreading of multidrug resistant TB worldwide. Given that TB is a disease which often involves more than one member in a family, a closer collaboration is needed between pediatricians and clinicians who take care of adults.

## 

The aim of this review is to provide updated knowledge on childhood tuberculosis (TB), emphasizing the peculiarities in transmission, natural history, clinical features, diagnosis and treatment of TB in children. Knowledge of clinical peculiarities of pediatric TB is now very important because of its ongoing spreading in poor resource countries and its recently increasing incidence in high resource countries [1,2]. This event has been associated to immigration of individuals from high-endemic areas (such as Asia, Africa, South America and Eastern Europe) to low-endemic countries (i.e. Western Europe and United States), as well as to lack of surveillance systems in low-endemic countries in the last decades. On the other hand, improved diagnostic strategies in high resource countries favour early TB recognition in children. The World Health Organization (WHO) estimated that 8.7 million cases of TB occurred worldwide in 2011 [3].Most of the TB cases in 2011 occurred in Asia (59%) and Africa (26%).

A smaller number of cases are reported in the Eastern Mediterranean region (7.7 %), in the European region (4.3%) and in the region of the Americas (3%). It is estimated that pediatric cases account for 10-15% of the global TB cases and that the majority of them occur in infants and children under the age of 5 years [3,4]. Despite the importance of pediatric TB, there is very little information about the epidemiology of TB in children worldwide, since WHO data concerns only smear-positive children [5,6].

## Differences in transmission

It has long been reported that children with TB are less contagious than adults, because they often have a paucibacillary disease, are less likely to have cavitary lesions in lungs and have a less forceful cough as compared to the subsequent ages [7-9] . Singh *et al.* reported the prevalence of TB infection in children who, in their household, came into contact with adults with pulmonary TB disease and shown a significant difference in contagiousness between smear-positive patients and smear-negative ones [9]. Transmission occurred globally in 95 out of 281 children (33.8%), of which 65 (68.4%) were contacts of sputum-positive adults whereas only 30 (31.6%) were contacts of sputum-negative ones [9]. The rate of TB transmission from smear-positive adults seems to be very high (from 40 to 60%), even if TB transmission from smear-negative subjects with active TB has been clearly documented [10-14]. A limited number of studies reporting rates of *M. tuberculosis* transmission from a pediatric case index are available in literature (Table 1) [15-25]. Most of these involve adolescents and describe school outbreaks [15-19]. The rate of TB transmission to contacts of smear positive adolescents varies from 7 to 39% [15,16,18] whereas transmission to contacts of smear positive younger children (7-10 years old) goes from 2 to 20% [20-22]. Only Molicotti *et al.* described an high risk of TB transmission from a child aged 10 years, founding an interferon-gamma release assay (IGRA) positive in 21 (72%) of 29 contacts [23]. However, the limited number of contacts in this report and lack of information on microscopy on respiratory specimens from the source case do not add consistent data to this issue. Few reports are available if the source case is a young child and they involve infants in hospital, accounting for a transmission rate from infants of 0-3% [24,25]. No data were available about transmission rates from sputum-negative children, but two recent reports from Paranjothy *et al.* and Baghaie *et al*. showed an extensive transmission from two smear negative children aged 9 and 15 years, showing that transmission of *M. tuberculosis* from smear-negative children is possible [17,19]. However it is not clear if contacts involved in these reports have some other risk factors for TB, for example being household contacts of adults with TB [18,20].

**Table 1:** Transmission rate of tuberculosis from pediatric index cases[15,16-25]. n.a. = not available

Index case (age/smear)	Transmission rate to pediatric contacts n (%)	Transmission rate to adult contacts n (%)	Transmission rate to contacts n (%)	Reference
9 years Smear positive	n.a.	n.a.	56/27 (20%)	Curtis et al, 1999 [20]

7 years Smear positive	22/169 (13%)	19/49 (38,7%)	41/218 (18,8%)	Cardona et al, 1999 [21]

4 months Smear positive	0/44 (0%)	1/142 (0,7%)	1/186 (0,5%)	Ciofi degli atti et al, 2011 [25]

7 years Smear positive	1/16 (6,7%)	4/211 (1,9%)	5/227 (2,2%)	Lee et al, 2005 [22]

10 years n.a.	21/29 (72,4%)	n.a.	21/29 (72,4%)	Molicotti et al, 2008 [23]

16 years Smear positive	67/765 (8,7%)	0/172 (0%)	67/937 (7,1%)	Caley et al, 2010 [18]

9 years Smear negative	85/200 (42,5%)	n.a.	85/200 (42,5%)	Paranjothy et al, 2008 [19]

4 months Smear positive	n.a.	n.a.	17/525 (3,2%)	Reynolds et al, 2006 [24]

15 years Smear positive	58/559 (10,3%)	7/67 (10,4%)	65/626 (10,3%)	Phillips et al, 2004 [15]

13 years Smear positive	195/486 (40%)	12/40 (30%)	207/526 (39,3%)	Sacks et al, 1985 [16]

15 years Smear negative	20/52 (37,7%)	0/15 (0%)	20/67 (29,8%)	Baghaie et al, 2012 [17]

Finally, a recent systematic review by Roberts *et al.* came to the conclusion that transmission to close contacts in school outbreaks are higher if the source case is a child 3 to 11 years of age rather than an adult [10]. In summary, since details on children’ own risk factors and type and duration of contact with the source case are lacking in the majority of these reports, further studies are necessary to elucidate contagiousness of young children in the community.

## Clinical peculiarities

### Natural history

Infection with *M. tuberculosis* can lead to a variety of outcomes which are generally different in children and adults. In the pediatric age the time between infection and the development of symptomatic disease is often very short. Without an adequate treatment, the risk of progression to active disease has been estimated to be higher in infants (30-40% in those younger than 1 year of age) and children (24% in 1-5 years of age) as compared to the subsequent ages. The risk of progression increases again in adolescence (15%), whereas in adults is about 5-10% [6]. Following the inhalation of *mycobacteria*, innate immunity controls infection in immune-competent patients. Tubercle bacillus initially lodges in the lungs and immunity prevents dissemination. The enlarged regional lymph node on chest radiograph, which may be associated with Ghon focus, is the hallmark of primary TB. In this phase children are often asymptomatic or have unspecific respiratory signs. Tubercle bacillus can multiply and the infection can progress to a caseating lesion and mycobacteria can spread, leading to haematogenous dissemination (meningitis or miliary disease). This occurs more often in infants and young children than in immune-competent adults [26,27]. Children are in fact prone to develop extra-pulmonary TB; about 4% of children infected under the age of 5 years, in fact, develop tubercular meningitis or miliary TB [27].

### Latent TB infection (LTBI)

LTBI is a very old definition, introduced in 1909 from von Pirquet who observed positive tuberculin reactions in children who did not have any TB clinical features [28]. Since that time, LTBI indicates a subject with TB infection without progression to active TB. Accordingly to international guidelines LTBI is therefore commonly diagnosed in asymptomatic children who have a normal chest X-ray (or with evidence of healed infection) and a positive tuberculin skin test (TST) or IGRA result [29,30]. Most guidelines, in the past, recommended to consider positive any TST result > 10 mm diameter (or any result > 5 mm if recently exposed to TB or immunocompromised), independently from vaccination with BCG. Recently, at the light of new evidences, UK guidance on TB recommend to consider a TST positive if induration is > 6 mm diameter for non vaccinated and > 15 mm for Bacille Calmette-Guerin (BCG) vaccinated subjects [30]. Moreover, recently, the concept of LTBI has been partially revised. LTBI was considered to be associated with a low number of dormant mycobacteria and the subject was considered to be infected, but not ill nor infectious. There are now some evidences that LTBI is a quite complex clinical condition, with a dynamic equilibrium between latency (characterized by absence of mycobacterial replication) and active disease [31]. This may be particularly true in childhood when, in recently exposed children, the boundary line between primary TB and LTBI may be hardly defined.

### Pulmonary tuberculosis

Pulmonary TB includes intrathoracic lymphadenopathy and parenchymal disease. In children, the infection in the lung is often characterized by a Ghon focus with regional lymphadenitis, called “primary complex”. Primary TB can rapidly progress, especially in younger children, to lung tissue destruction and a caseating cavity formation (progressive primary TB). Instead in adults, active pulmonary TB more frequently represents a secondary reactivation of a latent TB infection [32].Primary focus is subpleural in 70% of cases and in 25% of cases the foci are multiple. The lesions usually affect the lobes with greatest ventilation, i.e. the right upper and middle and the left upper lobe [33]. Children can develop a lobar pneumonia and the colliquative necrosis of the lung parenchyma may lead to the formation of a cavity. Calcifications in children are not commonly seen on chest X-rays. The presence of calcifications indicates that the lesion has been present for at least 6-12 months [34,35]. Pleural involvement may be due to a direct spread of caseous materials from a subpleural lesion or lymph nodes [32], but it is also thought to be the result of a hypersensitivity reaction to mycobacterial antigens. Detection of *M. tuberculosis* in pleural fluid is therefore possible in only about 56% of children with TB, but biopsy of pleural tissue has a higher culture yield [35]. Pleural TB is uncommon in children under the age of 6 years, and it is rare in those younger than 2 years of age; instead, it has commonly been observed in adolescents [32,36]. The most common clinical features of pulmonary TB in young children are non productive cough, low fever and, in rare cases, weight loss. Wheezing, which is usually secondary to extrinsic compression by ilar lymphonodes or even to lumen reduction by endobronchial granulomas, may occur particularly in younger children because of the small airways caliber [37].An unusual clinical finding, especially in low resource countries, is croup, due to a laryngeal TB with granulation tissue of the glottis. The infection of the glottis can be either due to the direct spread from the bronchus to the larynxor to hematogeneous spread [38]. Gie *et al.* reported that among 354 children initially suspected of suffering from TB, 71 (20%) were found to have other pulmonary diseases such as pneumonia (29%), bronchopneumonia with wheezing (18%) ad asthma with lobar or segmental collapse (12%) [37].

Radiological evidences of pulmonary TB in children are less specific than in adults [39]. Adult type disease is distinguished by cavitation that occurs predominantly in the lung apices, in the posterior segment of the upper lobes and in the superior segments of the lower lobes. In younger children the most common radiological findings are non specific lesions and the most typical feature is hilar or paratracheal lymphadenopaty [40]. Adult type disease may occur in adolescents, who usually develop it within 2 years after primary infection [39]. A study carried out in Nigeria investigated the radiological features of TB in 423 patients including 47 children. The most common radiological features in adults were cavitary formations, streaky opacities and nodular opacities, whereas the most common findings in children were hilar lymphadenopathy, bronchopneumonia and pleural fluid collections [40]. The enlargement of lymph nodes is not often visible in chest X-ray, partially because of the presence of the thymus, and the chest X-ray may appear normal. A recent study carried out in asymptomatic children with TB infection has reported that the frequency of chest X-ray abnormalities was only 1,8% [41]. Therefore, differential diagnosis between active and latent TB are not always easy and a computed tomography (CT) scan with contrast may often be helpful. Enlarged lymph nodes may be detected by CT scan in 60% of children with normal finding on chest X-ray [42].

### Tubercular lymphadenitis

The most common manifestation of extrapulmonary TB in the pediatric age is tubercular lymphadenitis. This is part of the primary complex and in children it usually develops within 6 months from infection [27]. More common than in adults, the enlargement of regional lymph nodes as result of TB usually occurs in response to a pulmonary focus within their drainage area and it generally involves cervical, submandibular, supraclavicular, preauricular or submental areas [27,43].

### Central Nervous System (CNS) tuberculosis

The most severe manifestations of extra-pulmonary TB in children, as well as in adults, are those occurring in the CNS. Tubercular meningitis (TBM) is more common in early childhood, whereas tuberculoma is usually (but not exclusively) found in older children and adults. Uysal *et al.* recently reported that, excluding the lymph nodes, the most commonly involved site in childhood extra-pulmonary TB is the CNS [44]. Even with anti-tubercular therapy, TBM short-term mortality is high, ranging from 20 to 69% with permanent neurological sequelae occurring in 50% of cases [45,46].

TBM occurs in children more frequently within 2 years of primary infection, predominantly in infants under the age of 5 years [47,48]. The onset is often gradual with non-specific symptoms and diagnosis is consequently difficult. In a recent study conducted in adult and pediatric patients with TBM, the most common signs and symptoms were headache (69%), fever (69%), nausea/vomiting (61%), anorexia (54%), altered mental state (58%),weight loss (37%), cough (33%) and drowsiness (28%) [49]. Tuberculoma typically occurs in cerebellum in children and in the frontal lobe in adults [27]. The presenting signs are essentially those of a space-occupying lesion: headache, nausea/vomiting and focal neurological signs, and they may differ in children and adults because of the typical different localization of tuberculoma [50-52].

### Bone tuberculosis

Bone and join lesions are more frequent in childhood than in adult age and occur up to 5% of all pediatric TB cases [27]. Skeletal TB usually is a disease of the older child, with the exception of spinal involvement, which can occur even in younger children. The most common manifestations of skeletal TB are osteomyelitis, spondylitis and arthritis [32]. Osteomyelitis can occur in any bone and can be multiple but tuberculous dactylitis and thoracic and lumbar spinal TB (Pott’s disease) are the most common manifestations in young children. Pott’s disease accounts for up to 40% of bone TB in children, whereas it is rare in adult population [27]. Moreover, vertebral destruction is more severe in children than in adults because bones are mostly cartilaginous [53]. However, *M. tuberculosis* does not produce proteolytic enzymes that can destroy cartilage. Hence there is potential for preservation of good function when early diagnosis is made[27]. Usually immune-competent children have solitary lesions, whereas multiple lesions are common in immune-compromised pediatric patients [32].

All the clinical manifestations listed above are the more frequent ones observed in pediatric TB. Less common manifestations are summarized in Table 2[53-72]

**Table 2:** Clinical manifestations of tuberculosis in children [53-72]

Area involved	Clinical presentation
**More common**	

Lungs and airways	Pneumonia, cavitary lesions, wheezing, laryngeal involvement

Lymph nodes	Enlargement of mediastinal, cervical, submandibular, supraclavicular, preauricular, submental and abdominal lymph nodes

Central nervous system	Meningitis, tuberculoma

Bones and skeletal muscles	Pott’s disease, arthritis, cystic of bone, abscess of skeletal muscles

**Less common**	

Abdomen	Pneumatosis intestinalis, peritonitis, liver and splenic abscess, enterolithiasis, intestinal perforation

Genitourinary tract	Scrotum inflammation, hydrocele, calyceal destruction, ureteral strictures, small-capacity bladder, hydronephrosis, kidney calcification

Heart and vessels	Intracardiac tuberculoma, pseudoaneurysms

Oral cavity	Enlargement of the tonsils, rethropharyngeal abscess, granulomatous cheilitis

Eyes	Uveitis, episcleritis, optic neuritis, orbital tuberculoma

Skin	Scrofuloderma lesions, lupus vulgaris, tuberculosis verrucosa cutis

## Diagnostic peculiarities

Diagnosis of TB in children is commonly obtained using the Tuberculin Skin Test (TST) and Interferon Gamma Release Assays (IGRAs), associated to radiological techniques. However, the gold standard for diagnosis of active TB is the detection of *M. tuberculosis* clinical specimens. IGRAs are immunological tests which are based on the important role that interferon-gamma plays in immunological response to *M. tuberculosis*. They are extensively dealt with in a dedicated chapter in this supplement.

### Tuberculin Skin Test (TST)

TST available are the Mantoux test and the multi-puncture test, but only the Mantoux test is a standardized method. Hence, the Mantoux test is the preferred test for detecting infection by *M. tuberculosis* and it is currently reported as a synonym of TST. A positive TST reaction is a hallmark of TB infection, but it does not distinguish between latent infection and disease. Tuberculin reactivity usually becomes apparent in 6 weeks, but occasionally can take up to 3 months after infection [73].

False-negative results in adults may occur in patients with immunosuppressive illness, malnutrition, viral infections (such as Human Immunodeficiency Virus infection, influenza, measles and chickenpox) and active disseminated TB. False-negative results in children can be due to the same conditions or only to the young age. Sensitivity is lower in children than in adults. Two recent meta-analysis reported a 80-82%sensitivity of TST in active disease in children [74,75]. By contrast, other authors found a 94%sensitivity in adults [76]. Therefore, after a recent exposure to a source case of TB, a negative TST result does not exclude the diagnosis of TB, particularly in young children. In adults and children who are older than 5 years, a negative result obtained <8 weeks after exposure is considered unreliable for excluding infection and a follow-up test at the end of the window period is therefore necessary. In children under the age of 5 years, the low sensibility of TST and the higher risk of progression of TB infection justify a more timely therapeutic attitude. So far, in this age group, treatment for suspected M. tuberculosis infection (window prophylaxis) is recommended also if the initial skin test induration diameter is <5 mm. Only after a second TST administered 8 weeks post-exposure (and absence of clinical and radiological abnormalities) the decision to treat or not to treat is reconsidered [77]. False-positive TST can result from vaccination with (BCG) or after contact with non tuberculous mycobacteria (NTM), since the purified protein derivative (PPD) contains a mixture of over 200 antigens including some antigens common to BCG and NTM [78,79].

### Detection of M. tuberculosis in sputum or gastric aspirate

The gold standard for diagnosis of active TB is the detection of *M. tuberculosis* in clinical specimens. Sputum microscopy is the most useful test in adults for diagnosis worldwide even though it has a sensitivity of 60% or less, mainly because a positive smear requires at least 5000-10000 acid-fast bacilli (AFB) per μL sputum sample. An added value of smear microscopy, thanks to the semiquantitative score, is its possible use for therapy monitoring. Culture for *M. tuberculosis* takes 8 weeks to be considered negative but following the implementation of liquid media, on overage 80% of positive results are reported in less than 20 days. It requires only 10-100 AFB per μL, and can detect pulmonary TB in around 80% of cases [80]. Nucleic acids amplification can provide results within 24-48 hours and performs well on smear-positive specimens with a sensitivity close to 100%; the sensitivity is in contrast suboptimal on smear negative sample [81,82].

Detection of *M. tuberculosis* in sputum is even more difficult in children for at least two reasons. Firstly, as young children are frequently unable to expectorate, a number of procedures have been suggested to obtain samples from the lower respiratory tract. The collection of three consecutive early morning gastric aspirate samples, is widely used with the aim of recovering swallowed sputum for microbiological confirmation in children. A number of less invasive alternative methods have been recently proposed, including induced sputum, nasopharyngeal aspiration and the string test. Secondly, because of the paucibacillary nature of TB disease in children, smears from sputum or from gastric aspirates are positive in less than 15% of children diagnosed with TB and a positive culture is achieved only in less than 40% of cases and even lower is the sensitivity of the nucleic acids amplification [83]. Therefore, investigations in sputum or in gastric aspirate can have a poor performance in identifying active TB in children [84,85]. Microbiological diagnosis of different forms of extrapulmonary TB does not substantially differ in children and adults. Cerebrospinal and lymphonodal are the most frequent extrapulmonary TB localizations in children. The diagnosis of cerebrospinal TB is challenging mainly in consequence of the extremely low number of bacilli due to the limited volume of cerebrospinal sample usually available, its subdivision between multiple tests is often self defeating and the highest sensitivity is achieved using the whole sample for the culture in liquid medium only. The nucleic acid amplification tests too may present reasonable sensitivity, at least with systems accepting large volume of sample (up to 2 mL) [86]. The microbiological diagnosis of lymphonodal TB is not problematic once a proper sample (fine needle aspirate or biopsy) is available. In this case, in particular in presence of cervico-facial localization, the differentiation of tubercular from non-tubercular etiology is crucial and may be confidently achieved by resorting to methods based on reverse hybridization, which are commercially available.

Because of the increase of drug-resistant TB the susceptibility testing is essential for every newly diagnosed case. The phenotypic test, which is the gold standard, performs well in particular for the major drugs rifampicin and isoniazid; it takes on overage two weeks once the strain has grown in culture. In presence of Multi-Drug-Resistant TB (MDR-TB) the antimicrobial susceptibility testing to second line drug is needed; because of standardization problems it should be performed by reference laboratories only. In the last years a number of mutations have been detected which are responsible for resistance to different drugs. The recognition of specific mutations in certain genetic regions is predictive of resistance. Commercially available molecular methods can detect mutations responsible of resistance on strains grown in culture or directly on smear positive clinical specimens. They are able to detect rifampicin resistance in more than 95% of rifampicin-resistant strains [87]. Rifampicin resistance is often considered a surrogate marker of MDR-TB as about 90% of resistant strains are resistant to isoniazid too. Methods suitable to detect mutations responsible of resistance to other drugs are also offered, although very specific, they are characterized by sensitivity clearly lower than that of rifampicin [Table 3].

**Table 3:** Gene investigated for mutations responsible of resistance to different drugs and sensitivity percentage of resistant strains presenting mutation in the particular gene [85].

Drug	Gene arbouring mutations	Sensitivity
Rifampicin	*rpoB*	> 98%

Isoniazid	*katG*, *inhA*	80-90%

Quinolones	*girA*	70-90%

Amikacin, Capreomycin, Kanamycin	*Rrs*	50-90%

Ethambutol	*embB*	70-80%

In summary, there is a need for improved accuracy of the diagnosis of TB in children. In adults smear and sputum culture provide reliable standards because of the diagnosis is usually confirmed microbiologically [66]. In contrast, in children there is a lack of a standardized TB case definition and epidemiological, clinical and radiological findings are always needed to make diagnosis of TB. Some experts in pediatric TB have recently proposed clinical case definition categories (confirmed, possible, probable, unlikely, or not TB) for intrathoracic disease in children. Children with confirmed TB are those who have at least one sign or symptom suggestive of TB and microbiologically confirmed TB. Children with probable TB are those who have at least one sign or symptom suggestive of TB and a suggestive chest radiography and a positive clinical response to anti-tuberculosis treatment or a documented exposure to *M. tuberculosis* or immunological evidence of *M. tuberculosis* infection. Children with possible TB are those who have at least one sign or symptom suggestive of TB and a suggestive chest radiography or a positive clinical response to anti-tuberculosis treatment or a documented exposure to *M. tuberculosis* or immunological evidence of *M. tuberculosis* infection. Children with unlikely TB are those with only signs or symptoms suggestive of TB. Children in whom TB may be excluded are those without signs or symptoms suggestive of TB and with an alternative diagnosis [88].

## Treatment of TB

The recommended treatment regimens for latent and active TB are generally the same for children as for adults. Treatment of latent TB infection is always necessary in children, instead it is not recommended in adults aged more than 36 years because of the increasing risk of hepatotoxicity of drugs with age and because of latent TB in adults is rarely due to a recent infection. Treatment is necessary if adult patients are infected with HIV (because of the major risk of active TB development) or if they are healthcare workers (because of the risk of transmission of *M. tuberculosis* among patients) [89]. Treatment for latent TB involves the use of one or two anti-tubercular agents to prevent the future development of TB disease. Three regimens, containing isoniazid or/and rifampicin, are now recommended for treatment of latent TB in children by the international guidelines [89-93]. Randomized trials have shown that treatment with isoniazid is highly effective, with 90% protection provided by completion of 9 months and 60-80% protection provided by completion of 6 months in adults [90,93]. In children, treatment with isoniazid for 9 months provides protection to approximately 100% . Adverse events associated with isoniazid are generally mild, such as nausea or headache, but can include rashes, hepatitis and peripheral neuropathy. The most serious complication is isoniazid-induced hepatitis, which can progress to fulminant hepatic failure and death [94]. Also treatment with rifampicin for 4 months is recommended in adult population and for 6 months in childhood [89-91]. Although not recommended by all the international guidelines, the 3-month regimen of both rifampicin and isoniazid is often used as alternative regimen in adults and children [90,92]. In a meta-analysis of four small randomized trials, this regime appeared to provide 60% protection in adults but in children data about its effectiveness are not available [94].

Isoniazid, rifampicin, pyrazinamide and ethambutol are the first-line drugs in the treatment of active TB disease in adults and in children. The most common regime used is a two-month intensive phase of daily isoniazid, rifampicin and pyrazinamide followed by a four-month continuation phase of daily isoniazid and rifampicin. In the first two months, the addition of a fourth drug such as ethambutol or a second-line anti-tubercular drug is necessary in complicated pulmonary TB or in meningeal involvement and, in addition, can protect against the emergence of drug resistant organisms [89].

Ethambutol was not recommended for use in children aged less than 5 years because optic neuritis (the most common form of toxicity which can lead to irreversible blindness) is difficult to monitorize in younger children [95]. Recent studies have shown that ocular toxicity occurs in children taking high or prolonged doses of ethambutol. Thus, if the recommended dosages are adhered to it is now considered safe in children. In fact, a review of Donald *et al.* reported that among 3811 children who had received ethambutol, only two (0,05%) have developed ocular toxicity [83]. Therefore, screening children for ethambutol ocular toxicity seems unnecessary [97].

Until recently, recommended dosages in mg/kg for antitubercular agents in children were deducted from dosages used in adults. Dosages of first-line anti-tubercular drugs in children have been increased in 2009 by WHO, because several pharmacokinetic studies have shown that infants and young children have lower peak serum levels than adolescents and adults, because of a different rate of metabolism, clearance and distribution of drugs [98,99]. This concept has been recently confirmed by Verhagen *et al.* in a pharmacokinetic study coming from Venezuela and involving children younger than 16 years of age [100]. In addition, children tolerate anti-tubercular agents better than adults, partially because of a lower prevalence of underlying liver disease and alcohol usage [101]. A recent study has reported that these doses are safe also in children under the age of two years, in whom few studies on first-line drug pharmacokinetic are available [102]. The doses of first- and second-line anti-tubercular drugs recommended by the most recent international guidelines are listed in Table 4. The incidence of anti-tubercular drug-induced hepatotoxicity has been variably estimated between 2% and 28%, but studies concerning children are limited and include reports in which children and adults are evaluated together [103-105]. A recent review has reported that although anti-tubercular drug-induced hepatotoxicity can occur in children at any age or with any dosage of drugs, the incidence of it is considerably lower than in adults. In fact, among 12,708 children receiving chemoprophylaxis only one case of jaundice was recorded and abnormal liver functions were documented in 8% of the children studied [101]. In adults, older age seems to be a risk factor for hepatotoxicity, whereas younger children have been reported to be at lower risk [106,107]. However, children with disseminated forms of disease seem to be at greatest risk of anti-tubercular drug-induced hepatotoxicity [101].

**Table 4:** Doses of first- and second-line anti-tubercular drugs recommended in adults and in children [30,93,98,111].qd= ones a day; bid= twice a day; tid= three times a day.

Anti-tubercular drugs	Dosages in adults (mg/kg)	Dosages in children (mg/kg)
**First-line anti-tubercular drugs**		

Isoniazid	4–6qd	10–15 qd

Rifampicin	8–12 qd	10–20 qd

Pyrazinamide	20–30 qd	30–40 qd

Ethambutol	15–20 qd	15–25 qd

		

**Second-line anti-tubercular drugs**	**Dosages in adults** (mg/kg)	**Dosages in children** (mg/kg)

Capreomycin	15-30 qd	15-30 qd

Kanamycin	15-30 qd	15-30 qd

Amikacin	15-20 qd	15-30 qd

Streptomycin	12-18 qd	20-40 qd

Levofloxacin	7,5-10 qd	7,5-10 qd in children aged ˃ 5 bid in children aged ˂ 5

Moxifloxacin	7,5-10qd	7,5-10 qd

Ofloxacin	15-20qd	15-20 qd

Ethionamide	15-20 qd	15-20 qd

Protionamide	15-20 qd	15-20 qd

Cycloserine	15-20 qd	10-20 qd

Para-amino-salicylic acid (PAS)	150 qd	200-300 qd

		

**Anti-TB drugs with unclear efficacy**	**Dosages in adults** (mg)	**Dosages in children** (mg/kg)

Linezolid	600 qd	10 qd

Clarithromycin	500 bid	7,5 bid

Clofazimine	200qd	1 mg/kg qd

Meropenem	2000 tid	20-40 tid

Amoxicillin/clavulanate	2000/250 bid (maximum dosage)*	40 bid (based on amoxicillin component)*

* Recommended dosage of amoxicillin/clavulanate in adults and children is not clear because of the amoxicillin to clavulanate ratio in commercially available tables and oral suspensions goes from 2:1 to 7:1.

However, the emergency of MDR-TB has led to an increase of use of second-line anti-TB drugs and also of drugs with unclear effectiveness against *M. tuberculosis* both in adults and children [108]. Despite the fact that most of these agents were discovered many years ago, information is lacking regarding their pharmacokinetic and pharmacodynamic proprieties, adverse effects and drug interactions in children and therefore they are not formally approved for use in childhood by Food and Drug Administration. Given the increased incident of MDR-TB, to include children in trials of novel or existing agents is now a priority [109]. There are several barriers to include children in TB drugs development: infrequent transmission of TB from children, difficulty of confirming active tuberculosis among children, existent of effective therapy for drug susceptible TB, concerns about pediatric-specific side effects, uncertainties about the appropriate time to involve children in drug development, regulatory requirements engendered by the inclusion of children and concerns about further subdividing the limited resources available for drugs development [110]. Confirmation of MDR-TB may not be possible in children because of the paucibacillary nature of TB and information about the response to treatment of the index case is often needed. In addition, the second-line drugs are not available in pediatric formulations or appropriate table size and therefore dosing may be inaccurate and sub-therapeutic or toxic level are possible. Hence, although their use is often necessary because of the emergence of MDR-TB, carefulness is necessary in off-label use of these medications in children and more studies and multicenter trials should be conducted in children, to test the effectiveness and the safety of anti-tubercular second-line drugs [111]. Seddon *et al*. have recently demonstrated that hearing deficit is common in children treated with aminoglycosides and polypeptides and this can have profound implications for developmental of language. Children should be screened prior to begin treatment and then they should be monitor for hearing at least every month [112]. Ethionamide treatment instead may cause hypothytoidism as in developing children as in adults and the risk is higher for children on regimens including para-aminosalycilic acid and for HIV-infected children [113].

It is also important to note that ensuring compliance to treatment is a major challenge. This is even more true in children, whose major problems are the low palatability of medications and the daily pill burden. In fact, the daily pill burden can be vast in children because they may require multiple medications due to the few fixed dose drug combinations approved for pediatric age. Hence, good communication between the hospital team, general practitioner or pediatrician, patient and care givers is necessary to make the patient and the family understand that taking the medication is the better option [114].

The differences in treatment of TB between children and adults are summarized in Table 5, where the most important differences in transmission, natural history, clinical features and diagnosis of TB between pediatric and adult age are also listed.

**Table 5:** Summary of differences in tuberculosis between adults and children.

Category	Adults	Children
**Transmission**	are frequently contagious because they often have: - cavitary lesions - multibacillary disease - pulmonary tuberculosis - less circumscribed social networks - more forceful cough - productive cough	are infrequently contagious because they often have: - non-cavitary lesions - paucibacillary disease - extrapulmonary tuberculosis - more circumscribed social networks - less forceful cough - non-productive cough

**Natural history**	- risk of progression is 5-10% - time between primary infection and disease is often long (some years)	- risk of progression is: 45% in infants < 1 year of age; 24% in children 1-5 years of age; 15% in adolescents - time between primary infection and disease is often short (1-6 months)

**Clinical presentation**	- primary infection is often asymptomatic but symptoms and signs are specific - principally develop pulmonary TB	- primary infection is asymptomatic but it may rapidly progress to symptomatic TB disease with not specific symptoms and signs - often develop extrapulmonaryand military TB

**Diagnosis**	- for screening purposes TST or IGRAs are recommended - detection of *M. tuberculosis* in sputum smear is achieved in 80% of cases - chest radiography shows cavitary formations	- in children < 5 years of age only TST is recommended because IGRAs may be unreliable - in children ˃ 5 years of age and adolescents TST or IGRAs are recommended - detection in gastric aspirates of *M. tuberculosis* is achieved in less than 40% of cases - chest radiography shows unspecific lesions (e.g. hilar or mediastinal lymphadenopathy, bronchopneumonia and pleural fluid collections) or may be normal

**Treatment**	- treatment for latent TB in close contacts should be unnecessary - toxicity induced by anti-tubercular drugs is most common - use of second-line anti-tubercular agents is formally approved - fixed dose drug combinations are available	- treatment for latent TB is always necessary and in close contacts< 5 years it should be started also if TST is negative - toxicity induced by anti-tubercular drugs is less common (also ethambutol is considered safe in young children) - use of second-line anti-tubercular agents is not formally approved - few fixed dose drug combinations are available


TB= tuberculosis; TST= Tuberculin Skin Test; IGRAs = Interferon-gamma Release Assays.

## Conclusions

The recently increasing incident of TB in high resource countries has led to a rise of interest in this condition, particularly by pediatricians, because this finding has been especially observed in children. Hence pediatricians have to deal with an emerging disease, first considered almost disappeared and then little is known about it. In this review we provide information on childhood TB, emphasizing the most important differences in transmission, natural history, clinical features, diagnosis and treatment of TB between children and adults. Infection with *M. tuberculosis* can lead to a variety of outcomes, which are generally different between children and adults, and this confirms the well known concept that “the child is not a small man”.

The knowledge of peculiar clinical features of childhood TB allows a prompt diagnosis and a prompt treatment which are very important for at least three reasons. Firstly, children carry the greatest burden of developing disease and extrapulmonary involvement. In particular, tuberculous meningitis and miliary TB occur more often in children than in adults. Secondly, they represent the pool from which a large portion of future and contagious cases of adult TB will arise. Finally, because disease in young children reflects recent infection, rather than secondary reactivation as in adults, childhood TB may be considered as a marker of current transmission in the community and therefore the diagnosis of TB in children is also a public health problem and it should always lead to the research of a source case, which, considering that children are rarely contagious, is often an adult.

Despite its importance, prompt diagnosis of TB in children is more difficult than in adults because it is complicated by the low number of signs and symptoms in primary infection, the unreliability of IGRAs in children under the age of 5 years, the lower sensibility of TST in infants than in adolescents and adults, by the more difficult detection of *M. tuberculosis* in gastric aspirates than in sputum-smear in adults and by the non specific radiological findings of primary complex, which is more frequent in children than it is in adults. Hence, further research is needed to develop more sensitive tests for diagnosis of TB in children.

Promptness to make diagnosis allows early treatment with anti-tubercular agents, which greatly improves the prognosis of patients. Despite the risk of causing major side effects, such as drug-related hepatotoxicity, is less frequent in children than in adults, particular attention is necessary during the follow-up of the young patients to identify any adverse events of anti-tubercular drugs, particularly when they are treated with second-line anti-tubercular drugs for MDR-TB.

Moreover, given that TB is a disease which often involves more than one member of a family from different age groups, a closer collaboration is necessary between pediatricians and clinicians who take care of adults.

## Competing interests

the authors declare that have no competing interests

## Authors' contributions

PP, collected data from the literature, and drafted the manuscript. EC, ET, MDM and ET conceived the idea helped to draft and critically reviewed the manuscript.

## Declarations

Funding for this article came from the Italian Health Ministry /Young Research Project.

This article has been published as part of *BMC Infectious Diseases* Volume 14 Supplement 1, 2014: Highlights in Pediatric Tuberculosis. The full contents of the supplement are available online at http://www.biomedcentral.com/bmcinfectdis/supplements/14/S1
